# The Genetic Analysis and Clinical Therapy in Lung Cancer: Current Advances and Future Directions

**DOI:** 10.3390/cancers16162882

**Published:** 2024-08-19

**Authors:** Angela Rina, Debora Maffeo, Francesca Minnai, Martina Esposito, Maria Palmieri, Viola Bianca Serio, Diletta Rosati, Francesca Mari, Elisa Frullanti, Francesca Colombo

**Affiliations:** 1 Med Biotech Hub and Competence Center, Department of Medical Biotechnologies, University of Siena, 53100 Siena, Italy; angela.rina@student.unisi.it (A.R.); debora.maffeo@dbm.unisi.it (D.M.); maria.palmieri@dbm.unisi.it (M.P.); viola.serio@dbm.unisi.it (V.B.S.); diletta.rosati2@unisi.it (D.R.); elisa.frullanti@dbm.unisi.it (E.F.); 2 UOC Laboratorio di Assistenza e Ricerca Traslazionale, Azienda Ospedaliero-Universitaria Senese, 53100 Siena, Italy; francesca.mari@unisi.it; 3 Cancer Genomics and Systems Biology Laboratory, Department of Medical Biotechnologies, University of Siena, 53100 Siena, Italy; 4 Institute of Biomedical Technologies, National Research Council, 20054 Segrate, Italy; francesca.minnai@itb.cnr.it (F.M.); martina.esposito@itb.cnr.it (M.E.)

**Keywords:** lung cancer, genetic discoveries, target therapy, personalized treatments

## Abstract

**Simple Summary:**

Given its huge impact on global health, lung cancer remains a major diagnostic and therapeutic challenge. However, much has been achieved, and this review reports on recent advances, from the genetic understanding of lung cancer to personalized treatments and targeted therapies based on genetic landscape.

**Abstract:**

Lung cancer, including both non-small cell lung cancer and small cell lung cancer, remains the leading cause of cancer-related mortality worldwide, representing 18% of the total cancer deaths in 2020. Many patients are identified already at an advanced stage with metastatic disease and have a worsening prognosis. Recent advances in the genetic understanding of lung cancer have opened new avenues for personalized treatments and targeted therapies. This review examines the latest discoveries in the genetics of lung cancer, discusses key biomarkers, and analyzes current clinical therapies based on this genetic information. It will conclude with a discussion of future prospects and potential research directions.

## 1. Introduction

Lung cancer is a neoplasm that originates from the cells of the respiratory system, including bronchi, bronchioles, and alveoli. It usually presents as a mass that can grow to obstruct airflow or cause bleeding in the lungs or bronchi. Lung cancer is one of the leading causes of cancer mortality globally, and its high incidence and often unfavorable prognosis make it a significant public health issue. According to the latest statistics, lung cancer is responsible for approximately 2.5 million new cases and 1.8 million deaths per year, accounting for nearly 18% of all cancer deaths worldwide. This high mortality rate is largely due to the often late diagnosis and the aggressive nature of the disease [1,2]. In the United States, lung cancer is the most common cause of cancer death among men and has surpassed breast cancer as the leading cause of cancer death among women [2]. In Italy, approximately 35,000–40,000 new cases are reported annually per 100,000 inhabitants, with a mortality rate of 81 per 100,000 among men and 12 per 100,000 among women [1,2]. Its incidence is continuously increasing in industrialized countries due to associated risk factors, including tobacco smoking, exposure to toxic substances such as asbestos and radon, and air pollution [1,2,3,4,5,6,7,8]. Extensive case studies have demonstrated the relationship between lung cancer and smoking, and it is estimated that heavy smokers (more than 40 cigarettes per day) have a 60 times higher risk of developing the disease compared to non-smokers. This risk decreases with the number of years since smoking cessation [3,4,5]. Non-smokers are also at risk from secondhand cigarette smoke. Other causes include smog and air pollution produced by the combustion of petroleum derivatives, as well as processes involving the use of specific metals (nickel, chromium) and radioactive substances. Many occupational substances are recognized as lung carcinogens, although they are less significant than tobacco. Workers in tar, railways, and refineries are particularly at risk. Chemical substances tend to remain in the lungs for a long time due to their stability and difficulty in being eliminated [5,6,7,8].

**Figure 1:**
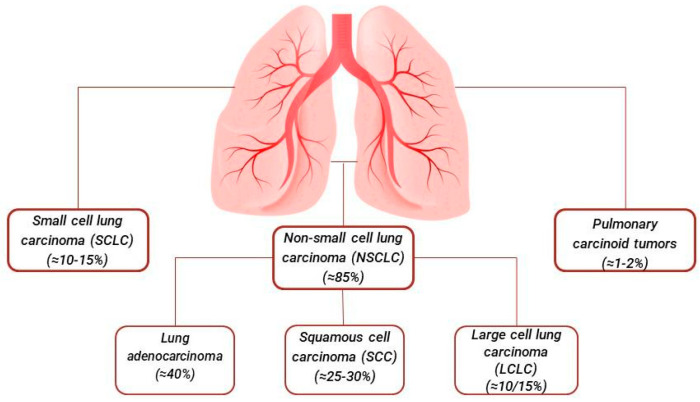
Classification of Lung Cancers. Lung cancer is divided into two main categories: about 85% of non-small cell lung carcinoma (NSCLC) including the subtypes of adenocarcinoma, squamous cell carcinoma (SQ) and large cell carcinoma (LC) and about 10–15% of small cell lung carcinoma (SCLC). Only 1–2% of lung tumors are pulmonary carcinoid.

Among the most implicated inorganic compounds is asbestos, followed by others with lesser frequency, including arsenic, chromium, nickel, and cadmium [8]. To date, the histological classification and staging of lung cancer by the World Health Organization (WHO) is constantly being updated and serves as the foundation for therapeutic advances, facilitating the development of targeted immunotherapies and ensuring precise diagnoses [9]. Epidemiological data on cancer provide essential information for the prevention, diagnosis, and management of the disease, supporting health measures. In the latest update, various advanced diagnostic methods have allowed for more accurate pathological and genetic classification of lung tumors, offering improved therapeutic options [9,10]. In general, lung cancer is primarily distinguished into two categories: non-small cell lung carcinoma (NSCLC) and small cell lung carcinoma (SCLC). NSCLC accounts for about 85% of cases and includes subtypes such as adenocarcinoma, squamous cell carcinoma (SQ), and large cell carcinoma (LC). Although less common, SCLC is known for its rapid growth and spread, accounting for about 10–15% of cases [9,10,11] Figure 1.

### 1.1. Lung Adenocarcinoma

Lung adenocarcinoma is a type of NSCLC that originates from the glandular cells of the lungs. It is the most common type of lung cancer, accounting for about 40% of NSCLC cases [2]. This tumor tends to develop in the peripheral areas of the lungs and is often diagnosed at an advanced stage, as it can be asymptomatic in the early stages [12]. The causes of pulmonary adenocarcinoma are multifactorial; cigarette smoking is the main risk factor, but this type of carcinoma can also affect non-smokers, with a higher incidence in women and young people [6,13]. Other risk factors include exposure to chemicals such as asbestos, radon, and air pollution, as well as a genetic predisposition [6,7,8]. The diagnosis of pulmonary adenocarcinoma requires a combination of clinical, radiological, and histological examinations. Imaging techniques such as chest X-rays, computed tomography (CT), and magnetic resonance imaging (MRI) are used to identify and evaluate the extent of the tumor [14,15]. Pulmonary biopsy, which takes a sample of tumor tissue for analysis, is essential to confirm the diagnosis and determine the specific characteristics of the tumor, including the presence of genetic mutations that may influence therapeutic choice [14,16].

The treatment of pulmonary adenocarcinoma depends on the stage of the disease and the general condition of the patient. Options include surgery, chemotherapy, radiotherapy, and targeted therapies. In cases where the tumor is localized and resectable, surgery offers the best chance for a cure [15,17]. Chemotherapy and radiotherapy are often used as adjuvant treatments to reduce the risk of recurrence [18,19]. Targeted therapies and immunotherapy are revolutionizing lung cancer treatment, offering new hope, especially for patients with specific genetic mutations [20,21]. The prognosis for patients with pulmonary adenocarcinoma varies greatly depending on the stage at which it is diagnosed. In cases of early diagnosis, long-term survival rates are significantly better. However, many patients are diagnosed at advanced stages, when treatment is more complicated and survival prospects are reduced [22].

### 1.2. Squamous Cell Carcinoma

Squamous cell carcinoma (SCC) is a type of NSCLC that originates from the squamous cells lining the airways of the lungs. It accounts for approximately 25–30% of all NSCLC cases and is strongly associated with tobacco smoking, being more common in men and long-term smokers [2]. This type of carcinoma often develops in the central airways and can cause bronchial obstructions. The main risk factor for pulmonary squamous cell carcinoma is cigarette smoking, responsible for the majority of cases [22]. However, exposure to chemicals such as asbestos, radon, and air pollution, as well as a family history of lung cancer, can also increase the risk [6,8]. Pulmonary squamous cell carcinoma is characterized by slower growth compared to other types of lung cancer, but it can still metastasize to other parts of the body [23]. Symptoms of pulmonary squamous cell carcinoma can vary depending on the location and extent of the tumor [22]. The most common symptoms include persistent cough, often with bloody sputum, breathing difficulties, chest pain, and recurrent lung infections such as pneumonia. Other symptoms may include weight loss, fatigue, and bone pain, especially if the cancer has spread to other areas of the body [24].

The diagnosis of pulmonary squamous cell carcinoma involves a combination of clinical, radiological, and histological examinations. Imaging, such as chest X-ray and computed tomography (CT), is used to visualize the tumor mass and assess its extent [23]. Bronchoscopy, which allows direct examination of the airways and tissue sampling, is often used to confirm the diagnosis through histopathological analysis of the sample [23,25]. Other tests, such as open lung biopsy or needle biopsy, may be necessary to obtain a definitive diagnosis [16,25]. The treatment of pulmonary squamous cell carcinoma depends on the stage of the disease and the patient’s overall condition. In cases where the tumor is localized, surgery to remove the affected part of the lung may offer the best chance for a cure [17,20]. Radiotherapy and chemotherapy are often used as adjuvant or neoadjuvant treatments to reduce tumor size before surgery or to eliminate any residual cancer cells after surgery. In advanced cases where surgery is not possible, chemotherapy and radiotherapy remain the main treatments [20]. Recently, targeted therapies and immunotherapy have shown promising results, offering new hope, especially for patients with specific genetic mutations or particular immune profiles [20,21]. The prognosis for patients with pulmonary squamous cell carcinoma varies significantly depending on the stage at which it is diagnosed [26,27]. Early diagnosis significantly improves the chances of successful treatment and long-term survival. However, since many patients are diagnosed at advanced stages due to the insidious nature of the initial symptoms, the prognosis generally remains reserved [25,26].

### 1.3. Large Cell Lung Carcinoma

Large cell lung carcinoma (LCLC) is a type of NSCLC characterized by the presence of large and anaplastic tumor cells that do not fall into the histological categories of squamous cell carcinoma or adenocarcinoma [28,29]. It accounts for about 10–15% of NSCLC cases and can manifest in any part of the lung, although it tends to develop more frequently in peripheral areas [9,30]. The primary causes of large cell lung carcinoma are similar to those of other types of lung cancer, with cigarette smoking being the main risk factor [31]. Exposure to carcinogens such as asbestos, radon, and air pollutants, as well as genetic predisposition, can also increase the risk of developing LCLC [6,7,8]. Despite its prevalent association with smoking, this type of carcinoma can also occur in non-smokers [6]. Large cell lung carcinoma is known for its rapid growth and ability to metastasize to other parts of the body, such as lymph nodes, liver, bones, and brain [32,33]. Symptoms of LCLC are often similar to those of other types of lung cancer and can include persistent cough, shortness of breath, chest pain, hemoptysis, and unintentional weight loss. Due to its aggressive nature, symptoms can progress quickly, often leading to a diagnosis at an advanced stage [33].

The diagnosis of large cell lung carcinoma requires a thorough clinical evaluation and a series of diagnostic tests. Imaging studies such as chest X-ray, computed tomography (CT), and magnetic resonance imaging (MRI) are used to identify the presence of the tumor and assess its extent [34]. Definitive diagnosis is obtained through a biopsy, which involves the removal of a tissue sample for histopathological analysis [16]. In some cases, bronchoscopy or CT-guided fine needle biopsy is used to obtain tissue samples. The treatment of large cell lung carcinoma depends on the stage of the disease at the time of diagnosis and the overall condition of the patient. In the early stages, surgery is the main treatment option and may include the removal of part of the lung (lobectomy) or the entire lung (pneumonectomy) [17,20]. However, due to the aggressive nature of LCLC, many patients are diagnosed at advanced stages when surgery is no longer feasible. In these cases, chemotherapy and radiotherapy are the primary treatment options. Recently, targeted therapies and immunotherapy have shown potential in the treatment of large cell lung carcinoma, offering new hope for patients, especially those with specific genetic alterations [20,21]. The prognosis for patients with large cell lung carcinoma is generally unfavorable, particularly because diagnosis often occurs at an advanced stage of the disease. Long-term survival depends on various factors, including the stage of the tumor at the time of diagnosis, the patient’s age and health conditions, as well as the response to therapies [32,33]

### 1.4. Small Cell Lung Cancer

Small cell lung cancer (SCLC) is a type of lung carcinoma that accounts for about 10–15% of all lung cancers [34]. It is characterized by rapid growth and an early tendency to metastasize [16,35]. This type of cancer is closely associated with tobacco smoking, with over 95% of cases diagnosed among smokers or former smokers [36]. The most common clinical manifestations at the time of diagnosis include the presence of central tumor masses, involvement of the mediastinum, and spread outside the chest in 75–80% of cases. Patients may present with symptoms such as cough, wheezing, difficulty breathing, coughing up blood, weight loss, pain, fatigue, and paraneoplastic syndromes [36,37,38]. SCLC is distinguished by the presence of small, round or oval tumor cells with scant cytoplasm and prominent nuclei. It is highly aggressive and tends to spread rapidly to regional lymph nodes and other parts of the body, including the liver, bones, and brain [37]. At the molecular level, SCLC is often associated with mutations in the TP53 and RB1 genes, which are crucial in regulating the cell cycle and preventing tumor growth. The loss of function of these genes contributes to the uncontrolled proliferation of tumor cells [36]. The diagnosis of SCLC is based on a multimodal approach that includes clinical evaluation, radiological imaging, and lung tissue biopsy. Computed tomography (CT) and positron emission tomography (PET) are crucial tools for staging and assessing the extent of the disease. Diagnostic confirmation is obtained through histopathological examination, which reveals the typical cytological characteristics of SCLC [39,40,41]. The treatment of SCLC depends on the stage of the disease at the time of diagnosis. Traditionally, SCLC is classified into two main stages: limited disease and extensive disease. For limited disease, the standard treatment consists of a combination of chemotherapy (often with etoposide and platinum) and thoracic radiotherapy [39,41]. This approach can potentially be curative in some patients. For extensive disease, the main treatment is systemic chemotherapy, as radiotherapy has a limited role. Recently, immunotherapy with immune checkpoint inhibitors, such as atezolizumab, has shown promising results when combined with standard chemotherapy [39,42].

### 1.5. Other Less Common Types of Lung Cancer: Pulmonary Carcinoid Tumors

Pulmonary carcinoid tumors are rare neuroendocrine cell neoplasms of the lung, representing about 1–2% of all lung tumors [9]. They are divided into typical carcinoids (TC) and atypical carcinoids (AC), with slower growth compared to other lung carcinomas [9,43]. The cause of pulmonary carcinoid tumors is not completely known. Unlike other lung tumors, they are not strongly associated with tobacco smoking. Genetic mutations, such as in the MEN1 and TP53 genes, may be involved [44]. Symptoms vary and can include persistent cough, hemoptysis, bronchial obstruction, and hormone secretion syndromes, such as carcinoid syndrome. Some tumors are asymptomatic [43]. Diagnosis is based on radiological imaging (CT, MRI) and histopathological confirmation through biopsy. Octreotide scintigraphy can identify somatostatin receptors on tumor cells [45]. Surgery is the main treatment for typical carcinoids, while for atypical carcinoids, chemotherapy or radiotherapy may also be necessary [46]. For advanced cases, immunotherapy and somatostatin analogs are considered [47]. The prognosis is generally favorable for typical carcinoids with a 5-year survival rate of 80–90%. Atypical carcinoids have a worse prognosis, with a 5-year survival rate of 50–70% [46].

This comprehensive review seeks to provide insights into the latest discoveries about the genetics of lung cancer, discusses key biomarkers, and analyzes current clinical therapies based on this genetic information, setting the stage for an open discussion on potential future perspectives and potential research directions. To do this, we systematically searched the Embase, MEDLINE, Web of Science, PubMed, and Google Scholar databases. Peer-reviewed articles from the last ten years were selected using the combination of the following keywords: “lung cancer genetics”, “targeted therapy lung cancer”, “EGFR mutations”, “ALK rearrangements”, and “immune checkpoint inhibitors”, without any restriction. The articles were chosen based on the relevance, quality, and innovativeness of the results.

## 2. Genetic Landscape of Lung Cancer

Lung cancer is a highly complex and heterogeneous disease with genomic diversity in each histological subtype, a complicated mutation spectrum, and a genetic regulatory mechanism that has not yet been elucidated [48]. It is increasingly evident that the genetic alterations in lung cancers are numerous but also complex and varied in terms of their timing and mechanisms. New findings highlight molecular lesions unique to each of the two primary lung cancer subtypes, as well as those shared by both. Some genes are implicated in both subtypes, while others are specific to one subtype and may influence its differentiation [49].

Identifying the genetic mutations that drive lung cancer progression is important to better understand the underlying mechanisms and to develop personalized therapies that target tumor cells and inhibit pathways that promote tumor growth and progression. The development and implementation of high-throughput genomic technologies, enabling the identification of key genetic mutations that drive cancer progression at a lower cost and with faster turnaround times than previously possible, has made it possible to have a more complete view of the mutations involved in lung cancer [50,51].

### 2.1. Main Driver Genes 

The main druggable genetic alterations in non-small cell lung cancer involved EGFR (epidermal growth factor receptor), KRAS (Kirsten rat sarcoma viral oncogene homolog), ALK (anaplastic lymphoma kinase), BRAF (V-raf murine sarcoma oncogene homolog B1), and ROS1 (c-ros oncogene 1) genes.

EGFR is a transmembrane protein with intrinsic tyrosine kinase activity, crucial for regulating cell proliferation, survival, and differentiation. Activating mutations in the EGFR gene were first identified in non-small cell lung cancers (NSCLC) in 2004, marking one of the most significant advancements in understanding the molecular biology of these tumors [52]. EGFR gene mutations in lung tumors are primarily found in exon 19 (deletions) and exon 21 (L858R substitution) [53]. Specifically, these mutations lead to constitutive activation of the receptor’s tyrosine kinase, independent of ligand presence, promoting growth, survival, and cell proliferation through downstream signaling pathways such as PI3K/AKT and RAS/RAF/MEK/ERK. EGFR mutations are more common in women, non-smokers, patients with adenocarcinoma histology, and Asian patients [54,55]. In these populations, EGFR mutation prevalence can reach up to 50%, whereas in Caucasian patients, it is in the range of around 10–15% [53,54]. The discovery of activating mutations of the epidermal growth factor receptor (EGFR) in patients with lung adenocarcinoma led to the development of a new family of biological agents, called tyrosine kinase inhibitors (TKIs), that have revolutionized the clinical management of LC patients.

Mutations in the KRAS gene are among the most common genetic alterations in lung cancers, particularly in NSCLC [56,57]. KRAS is a proto-oncogene that encodes a GTPase protein, which is involved in signal transduction that influences cell proliferation, differentiation, and survival. Under normal conditions, the KRAS protein alternates between an active (GTP-bound) state and an inactive (GDP-bound) state [57,58]. Mutations, however, result in a constitutively active protein that continuously stimulates downstream signaling pathways, such as the MAPK and PI3K-AKT pathways, promoting uncontrolled growth of cancer cells [56,57]. KRAS mutations are present in approximately 20–25% of NSCLC cases, with a higher prevalence in patients with adenocarcinoma compared to those with other histological subtypes, suggesting a strong correlation with tobacco exposure [55]. The most common types of mutations in lung cancers are primarily concentrated in codons 12, 13, and 61, with G12C, G12D, and G12V being the most frequent among these [59]. Each specific mutation can differently influence tumor biology and treatment response, generally leading to an unfavorable prognosis for the patient [60].

The ALK gene encodes a receptor tyrosine kinase that is involved in the regulation of cell growth. Originally identified in anaplastic large cell lymphomas [61], ALK alterations are now also recognized in various solid tumors, including lung cancers [62]. ALK mutations in lung cancers are often the result of genetic rearrangements, leading to the formation of fusion proteins with oncogenic activity [62]. In lung cancers, the most common rearrangement involves the fusion of the ALK gene with EML4 (echinoderm microtubule-associated protein-like 4). This rearrangement, known as EML4-ALK, was first identified in 2007 and results in the production of a fusion protein with constitutive tyrosine kinase activity, promoting cell proliferation and tumor survival [62,63]. This translocation accounts for about 5% of lung carcinomas, and among the multiple genetic alterations involved in the development of these tumors, it has emerged as an important biomarker [64]. In addition to the known rearrangement with EML4, other ALK fusion partners have been identified, including KIF5B, TFG, and KLC1 [65,66,67]. Although these rearrangements are less common, they still contribute to the pathogenesis of lung cancer through similar mechanisms of aberrant activation of the ALK pathway. ALK-positive NSCLC exhibits highly aggressive behavior and is often diagnosed at advanced stages compared to wild-type patients. 

Despite BRAF gene mutations in lung carcinoma being identified before EML4-ALK translocations, there have been few clinical studies completed on this type of lung carcinoma with BRAF mutation. To date, BRAF gene mutations are recognized as one of the genetic factors contributing to the development of various types of cancer, including lung cancer [68]. BRAF is a gene that encodes a serine/threonine kinase protein, which is part of the MAPK/ERK signaling pathway involved in the regulation of cell growth, differentiation, and survival [69]. Mutations in this gene can lead to abnormal cell signaling and, consequently, uncontrolled proliferation of cancer cells. Specifically, conformational changes occur in the BRAF protein, causing constitutive activation of the MAPK/ERK signaling pathway [70]. This persistent activation stimulates cell proliferation, inhibits apoptosis, and promotes the survival of cancer cells. Several BRAF mutations have been identified, the most common being the substitution from valine to glutamate at codon 600 (V600E), which accounts for over 90% of BRAF mutations in melanoma [71]. This mutation, in particular, creates a form of BRAF that is independent of upstream regulatory signals, leading to incessant kinase activity. BRAF gene mutations are present in a minority of lung cancer cases, with an estimated prevalence of about 1–3% in NSCLC. These mutations have been identified mainly in adenocarcinoma subtypes [72]. BRAF gene mutations can be classified into three main classes. Class I BRAF mutations are commonly identified in human tumors and represent about 50% of BRAF mutations in lung tumors [72,73]. They affect the V600 amino acid, including V600D/E/K/R, and act as active monomers independent of RAS, resulting in the marked activation of BRAF kinase activity and constant activation of the MAPK pathway [70]. Class II and Class III mutations, on the other hand, are non-V600 mutations and represent 50–80% of BRAF mutations in lung tumors [72]. Class II mutations are mainly found in the activation segment (such as K601, L597) or in the P-loop (such as G464, G469) [74,75], and have intermediate to high kinase activity. Class III mutations have been found in the P-loop (G466), the catalytic loop (N581), and the DFG motif (D594, G596) [74,75], and are characterized by absent or low kinase activity. In terms of prognosis, class I BRAF mutations generally show a slightly better prognosis compared to class II and III mutations. The latter are linked to more aggressive behavior, a less favorable clinical course, and earlier disease progression following initial chemotherapy. BRAF mutations globally exhibit a significant predominance among males (61%) and individuals who have a history of smoking (81%), with varying frequencies across different mutational classes.

The ROS1 gene encodes a receptor tyrosine kinase involved in the regulation of cell growth and intracellular signaling. Initially, it was found to be implicated in the development of human glioblastoma [76] and was later recognized in other malignant neoplasms, including lung, ovarian, and gastric tumors [77,78,79]. ROS1 gene rearrangements are described in 1–2% of patients with NSCLC and were first identified in 2007 [76,77]. The majority of recorded patients are young, light smokers, or non-smokers. These rearrangements are more frequent in adenocarcinoma but have also been reported in large cell carcinoma cases [80,81]. ROS1 mutations are primarily represented by gene translocations, where a part of the ROS1 gene fuses with another gene, creating a fusion oncogene. Among the most common translocations are fusions between ROS1 and the genes CD74, SLC34A2, TPM3, and SDC4 [77,80,82,83]. These fusions result in the production of potent oncogenic drivers that promote cell proliferation, activation, and cell cycle progression by activating downstream signaling pathways, accelerating the development and progression of lung carcinoma due to the upregulation of JAK/STAT, PI3K/AKT, and MAPK/ERK signaling pathways [84]. Despite the low frequency of ROS1 gene mutations, their identification is clinically significant as patients harboring this mutation tend to respond well to specific tyrosine kinase inhibitors [85]. This ensures that the therapeutic choice for the patient is targeted and precise.

### 2.2. Emerging Biomarkers

Lung cancer is a heterogeneous disease that requires precise patient stratification to optimize treatment options. In recent years, specific biomarkers such as PD-L1 (Programmed death-ligand 1), MET (Mesenchymal Epithelial Transition), RET, NTRK, PIK3CA, HER2 (human epidermal growth factor receptor), and STK11 have gained attention as crucial tools for the diagnosis, prognosis, and therapeutic management of lung cancer [86]. 

Programmed death-ligand 1 (PD-L1), also known as CD274, is considered an immune checkpoint that facilitates the suppression of the antitumor immune response. PD-L1 can be present on the surface of various cells, including macrophages, antigen-presenting cells, B and T lymphocytes, epithelial cells, muscle cells, and endothelial cells [87]. The factor inducing its expression is interferon-gamma (IFN-γ), released by CD8 T cells [88]. The receptor of PD-L1 (PD-1) is primarily expressed by activated cytotoxic T cells [87]. When PD-L1 binds to the PD-1 receptor on activated T cells, immune system suppression occurs [87,89]. This interaction prevents autoimmune responses in peripheral tissues during inflammations, contributing to maintaining immunological tolerance [90]. The ligand-receptor complex triggers two reactions that inhibit the immune response: the first is the inhibition of interleukin 2 (IL-2) synthesis [91], and the second is related to the inhibition of the T-cell receptor, known as the “stop signal”, which can modify the duration of contact between T cells and target cells or antigen-presenting cells [92]. The PD-L1-PD-1 interaction is exploited in carcinogenesis to evade the immune system [93]. Elevated levels of PD-L1 have been detected on the surface of various tumor cell types, including NSCLC. This mechanism allows tumor cells to escape the immune response; PD-L1 acts as a pro-tumorigenic factor by activating proliferative and survival signaling pathways, also promoting tumor progression [94]. In lung tumors, PD-L1 expression can be regulated by various mechanisms, including genetic mutations, gene amplifications, and transcriptional regulation induced by inflammation or hypoxia. PD-L1 overexpression has been associated with a worse prognosis and greater tumor aggressiveness [94]. Mutations in the PD-L1 gene in lung tumors are relatively rare compared to other mechanisms of overexpression. However, some mutations can significantly impact PD-L1 function and the immune response. Specifically, point mutations in PD-L1 have been found that can influence its ability to interact with PD-1, altering the antitumor immune response. Some studies have identified that these mutations lead to a loss of function or stabilization of the protein [95]. Additionally, amplifications of the 9p24.1 locus, where PD-L1 resides, have been observed in a fraction of lung tumors. These amplifications are often correlated with high PD-L1 expression and have been associated with an unfavorable response to PD-1/PD-L1 inhibitor therapies [96,97]. Finally, although rare, gene translocations involving PD-L1 can lead to the fusion of PD-L1 with other genes, influencing its expression and function. These translocations can generate chimeric isoforms with new functional properties for tumor cells [98]. Due to the clinical relevance in the context of immunotherapy observed in lung tumors, the molecular bases of PD-L1 regulation and its role in the tumor microenvironment are still being explored. The aim is to understand hidden molecular mechanisms to identify new therapeutic targets and treatment combinations that can improve patient outcomes [99].

The MET gene encodes a tyrosine kinase receptor known as hepatocyte growth factor receptor (HGFR). This receptor plays a crucial role in regulating cell proliferation, motility, morphogenesis, and survival [100]. Upon binding to its ligand HGF, MET undergoes dimerization and auto-phosphorylation, activating important intracellular signaling pathways such as PI3K/AKT and MAPK/ERK [101]. Alterations in the MET gene, including mutations, amplifications, and fusions, are implicated in the pathogenesis of various cancers, including lung cancers [100,101,102]. Point mutations in the tyrosine kinase domain of MET can lead to a constitutive activation of the receptor, promoting the growth and survival of tumor cells [103]. Among these, the most studied is the MET exon 14 skipping mutation (METex14), which prevents receptor degradation and results in prolonged MET signaling [104]. MET exon 14 skipping mutations occur in approximately 3–4% of NSCLC cases and are generally not associated with other driver mutations [105,106,107]. Amplification of the MET gene, detected in 1–6% of NSCLC patients, leads to an increased number of gene copies and overexpression of the MET receptor with aberrant signaling. This amplification is often observed in cases of acquired resistance to treatments with epidermal growth factor receptor inhibitors [107,108,109,110,111]. Recently, rearrangements of the MET gene, including KIF5B-MET fusion, have been identified and although rare, have shown significance in NSCLC. These fusions are present in approximately 0.2–0.3% of NSCLC cases and are considered potential driver mutations targetable by specific therapies [112,113,114]. In conclusion, MET gene alterations are associated with poor prognosis and resistance to standard treatments in lung cancers. Therefore, detecting these alterations is critically important for the therapeutic management of patients [115].

The proto-oncogene RET, first identified in 1985 [116], encodes a receptor tyrosine kinase that activates downstream signaling pathways (RAS/MAPK/ERK, PI3K/AKT, and phospholipase C-γ), thereby promoting cellular proliferation, migration, and differentiation [117]. Genetic alterations, including chromosomal rearrangements and point mutations, lead to aberrant RET activation, contributing to tumorigenesis. Specifically, chromosomal rearrangements of RET have been found in approximately 1–2% of NSCLC patients [83,118]. These alterations result in the formation of chimeric proteins with constitutive oncogenic activity, stimulating downstream signaling pathways that promote tumor cell proliferation and survival [119]. The most commonly identified rearrangement in NSCLC is the KIF5B-RET fusion [83,120,121], although other fusion partners such as CCDC6, NCOA4, TRIM33, and CUX1 have also been identified [122]. Patients with RET fusion-positive NSCLC represent a distinct molecular subgroup with specific clinical and pathological characteristics. RET gene fusions appear to be mutually exclusive with other mutations, including those in EGFR, KRAS, ALK, HER2, and BRAF genes, suggesting independent oncogenic driver roles. Furthermore, initial reports indicate that RET rearrangements are more frequent among younger (<60 years old), female, non-smoking patients with adenocarcinoma histology [83,120,121,123,124]. In addition to rearrangements, point mutations in the RET gene have also been identified in lung tumors, albeit less commonly than fusions. Similar to rearrangements, these mutations can lead to RET receptor activation, contributing to oncogenesis [125]. Therefore, RET gene mutations represent a significant class of oncogenic alterations in lung tumors, and understanding their molecular mechanisms is facilitating the development of new targeted inhibitors, advancing therapies for RET-positive patients.

Mutations in NTRK genes are emerging as significant molecular markers in lung cancers, influencing diagnosis, prognosis, and therapeutic options [126]. The NTRK1, NTRK2, and NTRK3 genes encode the TRKA, TRKB, and TRKC receptors, respectively. These transmembrane proteins belong to the TRK receptor family and play a crucial role in cellular signaling and neural growth by activating pathways such as PIK3/PLCγ/MAPK [127,128]. NTRK gene fusions are rare but frequent oncogenic alterations, present in up to 1% of all solid tumors [129]. In NSCLC, the frequency of these fusions is approximately 0.1–0.2%, occurring when the 3′ sequence of the NTRK gene fuses with the 5′ sequence of a fusion partner gene [130]. The resulting fusion protein is aberrantly expressed, constitutively activating the receptor’s kinase domain, which leads to persistent activation of cellular signaling pathways necessary for oncogenesis [129,131]. NTRK gene fusions typically exclude other canonical oncogenic mutations, suggesting they act as sole oncogenic drivers in the development and maintenance of their host tumors [132]. Most patients with lung cancers harboring NTRK gene fusions exhibit clinical characteristics similar to those with ALK, RET, or ROS1 fusions [130], and are often found in a younger population with minimal or no smoking history. However, studies have also identified NTRK gene fusions in patients of various ages and with a previous smoking history [130,133]. Additionally, many patients with TRK fusion-positive lung carcinoma have developed metastases in the central nervous system [134]. Generally, NTRK gene mutations in lung cancers can be associated with variable prognoses. Some studies suggest that patients with these mutations may benefit from targeted therapies with NTRK inhibitors, as these inhibitors block aberrant tyrosine kinase activity, thereby reducing tumor growth and improving clinical response [135]. Despite current knowledge, further studies are necessary to evaluate the exact incidence of NTRK gene mutations in different populations and to better understand the clinical implications of these mutations. These mutations represent a promising field for the personalization of therapy in NSCLC patients.

The PIK3CA gene encodes a catalytic subunit of phosphatidylinositol-3-kinase (PI3K), a class of enzymes involved in numerous cellular processes, including cell growth, proliferation, survival, and metabolism. Mutations in the PIK3CA gene have been implicated in various types of cancer [136], including lung cancer, with a frequency of 2–4% in NSCLC cases [136,137]. PIK3CA is often mutated or amplified due to a missense variant that mainly affects the helical binding domain (exon 9, E545K or E542K) or the catalytic subunit (exon 20, H1047R or H1047L) [136,138,139]. These alterations lead to constitutive and PI3K/AKT/mTOR pathway-independent PI3K enzymatic activation, resulting in uncontrolled proliferation and survival of cancer cells with consequent drug resistance [140]. PIK3CA gene mutations in lung adenocarcinomas have not been reported as mutually exclusive. On the contrary, co-occurrence with alterations in EGFR, BRAF, ALK, and more frequently, KRAS genes, has been observed. This observation raises the question of whether PIK3CA mutation alone can be a sufficient oncogenic driver for NSCLC tumor formation [141,142]. PIK3CA mutations have been reported to be associated with smoking exposure. Specifically, patients with a PIK3CA mutation exhibit greater smoking exposure than patients with an EML4-ALK translocation or EGFR mutation, and less smoking exposure than patients with smoking-associated aberrations such as KRAS [143,144]. Additionally, the PIK3CA mutation has been observed to occur more frequently in patients with various prior malignancies compared to NSCLC [144]. The presence of PIK3CA mutations in lung cancer has several clinical implications, including worse prognosis due to increased tumor aggressiveness and metastatic potential [140,143]. Furthermore, the PIK3CA/EGFR co-mutation has been associated with reduced efficacy of EGFR inhibitors, thus necessitating alternative or combinatorial therapeutic strategies [145]. This underscores the need for further studies to ensure a deeper understanding of molecular mechanisms to improve NSCLC treatment.

Mutations in the HER2 gene in lung tumors are a topic of growing interest in oncological research. HER2, also known as ERBB2, is an oncogene that encodes a tyrosine kinase receptor involved in the regulation of cell growth, differentiation, migration, and apoptosis [146]. Although HER2 alterations are well documented in various types of cancers, such as breast carcinoma, their role in lung tumors has only recently started to emerge [147]. HER2 mutations are relatively rare, found in approximately 1–3% of NSCLC, and are observed more frequently in adenocarcinomas compared to other NSCLC subtypes [125,146,147,148]. HER2 alterations in lung tumors can primarily manifest through two mechanisms: gene amplification and point mutations. Amplification of the HER2 gene leads to overexpression of the HER2 protein on the cell surface, promoting uncontrolled cell proliferation and tumor cell survival [149,150]. The point mutations described to date are insertions within a small stretch of exon 20 with A775_G776insYVMA insertion/duplication at the COOH-terminal end of the αC-helix; these can increase the receptor’s enzymatic activity independently of ligand presence, leading to constant proliferative signaling [147,149,150,151]. HER2 mutations in lung tumors are predominantly observed in female, non-smoking patients and are often associated with an unfavorable prognosis [147,148,152]. Patients with these mutations tend to present with more aggressive tumors and a less favorable response to standard therapies [151,152]. For this reason, it is necessary to further investigate HER2 mutations which, although rare, are emerging as mutations of particular interest, especially for the development and progression of adenocarcinoma. Further studies might indeed highlight HER2 as a promising and relevant therapeutic target.

Mutations in the STK11 gene, also known as LKB1, represent a tumor suppressor gene implicated in the autosomal dominant disorder that predisposes individuals to cancer known as Peutz–Jeghers syndrome (PJS) [153]. Located on chromosome 19p13.3, the STK11 gene encodes a protein that functions as a kinase involved in the regulation of energy metabolism, cell growth, apoptosis, and cell polarity. Disruption of these processes is implicated in carcinogenesis. STK11 mutations have been identified as significant genetic events in various cancer types, notably within the context of NSCLC [154,155]. STK11 mutations are recorded in approximately 20–30% of NSCLC cases, with a higher prevalence in adenocarcinoma subtypes compared to squamous cell carcinomas, and they deactivate the LBK1 protein [156]. LKB1 is a key regulator of the AMPK (AMP-activated protein kinase) pathway, a crucial pathway for cellular responses to energy metabolism and stress. It is one of the few known serine/threonine kinases to be inactivated, which implicates dysfunction of the AMPK pathway, leading to dysregulated cell growth and survival under energy-stress conditions [157]. This contributes to the uncontrolled proliferation of cancer cells. STK11 mutations are often associated with a more aggressive tumor phenotype and a poorer prognosis [158]. Additionally, STK11 mutations can influence therapeutic responses. For instance, tumors harboring STK11 mutations tend to exhibit intrinsic resistance to immune checkpoint inhibitors, such as anti-PD-1/PD-L1 antibodies [158,159,160,161]. This resistance may be due to the decreased presence of tumor-infiltrating lymphocytes (TILs) and a less inflammatory tumor microenvironment, defined as a “cold” immunosuppressive microenvironment [162,163]. Despite the complexity and critical nature of STK11 mutations, ongoing research continues to explore the interactions between STK11 and other molecular pathways to identify additional therapeutic targets and optimize treatment combinations.

A schematic representation which summarizes all genetic mutations, the frequency of their detection, what metabolic pathway they are involved in, and what subtype of lung cancer they are typical for, is shown in Table 1.

## 3. Therapies Based on Genetic Information

Lung cancer represents one of the leading causes of mortality worldwide. For many years, therapeutic options were limited to surgery, chemotherapy, and radiation therapy, which often had significant side effects and limited efficacy in advanced cases. With the advancement of genetic and molecular research, target therapies have emerged as new therapeutic strategies [164]. Targeted therapies are treatments that specifically act on certain molecules involved in the growth and proliferation of tumor cells. Unlike traditional chemotherapy and radiation therapy, which indiscriminately target all rapidly dividing cells, targeted therapies block specific molecular signals that promote tumor growth [165]. This approach not only improves treatment efficacy but also reduces side effects.

### 3.1. Targeted Therapy

Targeted therapies for EGFR gene mutations in lung cancers represent one of the most significant and well-studied innovations in the fight against NSCLC [166], including TKIs and monoclonal antibodies [166,167]. The concept behind TKIs is the use of small molecules capable of disrupting downstream signaling pathways that promote cell proliferation and survival. This effect is achieved either by directly inhibiting the kinase’s catalytic activity by interfering with ATP and substrate binding or by inhibiting receptor activation by blocking its dimerization [168]. Among these, the first-generation drugs targeting the EGFR tyrosine kinase domain were gefitinib and erlotinib [169,170]. Gefitinib binds to the ATP-binding site of the receptor, preventing autophosphorylation and receptor activation. It is used as a first-line therapy in patients with NSCLC and has shown significant improvements in progression-free survival (PFS) compared to traditional chemotherapy in patients with EGFR mutations [171,172]. Erlotinib inhibits EGFR tyrosine kinase like gefitinib, by binding to the ATP-binding region of the receptor and blocking autophosphorylation. It is used both as a first-line therapy and in patients with advanced or metastatic disease [171]. Also, Erlotinib has demonstrated significant improvement in PFS compared to chemotherapy in patients with EGFR mutations [173]. Among second-generation drugs, afatinib, for example, is an irreversible inhibitor of EGFR, HER2, and HER4 tyrosine kinases. Its ability to block multiple members of the ErbB family makes it effective against some forms of resistance developed with first-generation inhibitors [174,175]. Finally, among third-generation drugs, osimertinib, an irreversible inhibitor, is used for treating patients with activating EGFR mutations and the resistance mutation T790M, common in acquired resistance to first- and second-generation TKIs [176]. This has shown significant improvement in PFS and overall survival (OS) compared to first-generation TKIs, and it is particularly effective in patients with brain metastases [177]. This has made it a first-choice drug for patients with advanced disease [176]. A different approach to prevent EGFR activation and signaling is through monoclonal antibodies against TRK or their ligands. These are designed to interrupt the receptor signaling pathway upstream by neutralizing or blocking the ligand, receptor internalization, and via a cytotoxic action. Among those currently available are cetuximab, necitumumab, panitumumab, and matuzumab [178,179].

Most tumors harboring KRAS mutations have often been considered difficult to treat pharmacologically due to the frequency and heterogeneity of these gene mutations. However, two direct inhibitors of KRAS, sotorasib and adagrasib, have recently been identified as reliable and promising for treating patients with metastatic KRAS-positive lung cancer. Sotorasib is the first FDA-approved drug that irreversibly and selectively inactivates KRAS G12C, blocking the signaling pathway that promotes tumor growth. Phase 1 and 2 clinical trials demonstrated significant tumor reduction in patients with G12C KRAS-positive NSCLC, with an objective response rate (ORR) of about 37% and a median PFS of 6.8 months [180,181,182,183]. Adagrasib is another specific inhibitor for KRAS G12C currently in advanced clinical development. Like sotorasib, adagrasib binds irreversibly to the mutated protein, blocking cell proliferation. Preliminary clinical trial data showed a response rate similar to sotorasib, with an ORR of 45% in pretreated patients. The median PFS for adagrasib was found to be around 7 months. Additionally, it has shown an effective antitumor effect against brain metastases [184,185,186]. Besides specific inhibitors for KRAS G12C, research is expanding towards other KRAS mutations and associated signaling pathways. Indeed, combination therapies and the development of new drugs promise to further expand therapeutic options, improving survival and quality of life for patients with KRAS-mutated NSCLC.

Therapies for lung cancer with ALK gene mutations currently focus mainly on TKIs. Crizotinib was the first ALK inhibitor approved for the first- and second-line treatment of patients with ALK-positive NSCLC [187]. It showed significant improvements in PFS compared to traditional chemotherapy [188,189]. However, many patients develop resistance to crizotinib, requiring additional therapeutic options [190]. Indeed, ceritinib, a second-generation ALK inhibitor, has demonstrated efficacy in treating ALK-positive NSCLC in patients who have acquired resistance to crizotinib [191,192]. and in patients with advanced or metastatic cancer [193,194]. To date, numerous clinical trials are currently investigating additional ALK inhibitors, such as alectinib, brigatinib, lorlatinib, and therapeutic combinations to further improve outcomes in patients with ALK-positive NSCLC. The goal is to prolong PFS, enhance control of brain metastases, and increase overall survival.

In 2011, the United States approved Vemurafenib (PLX4032) as the first drug against cancer with BRAF mutation [195]. Introduced in 2008, Vemurafenib is a selective inhibitor of the oncogenic kinase B-Raf, targeting melanoma with the BRAF V600E mutation. This drug blocks the RAF/MEK/ERK pathway by inhibiting the activity of the V600E BRAF mutation [196]. In 2015, patients with BRAF-positive NSCLC treated with Vemurafenib showed a PFS of 7.3 months and an ORR of 42% [197]. Consistent with these findings, a phase 2 study in 2017 demonstrated that Vemurafenib improved PFS in previously untreated patients with V600 BRAF-driven NSCLC [198]. Dabrafenib, on the other hand, is a competitive adenosine triphosphate (ATP) inhibitor of BRAF specific for V600 mutations [199], while Trametinib is a MEK inhibitor, a downstream protein in the same signaling pathway [200]. These two drugs have been combined and have shown remarkable efficacy in patients with BRAF V600E-positive NSCLC [201]. Clinical studies have reported that the Dabrafenib–Trametinib combination results in an ORR of 64% and a median PFS of 10.9 months [202]; the high efficacy of this combination has led to the approval of this therapy as the standard of care for this subgroup of patients [203]. In addition to BRAF and MEK inhibitors, further studies are underway to develop new BRAF inhibitors and to explore the efficacy of new combinations with other therapies, which could further enhance treatment response and overcome emerging resistances.

The main targeted treatments approved for lung cancer with ROS1 rearrangement include drugs such as Crizotinib, a tyrosine kinase inhibitor initially approved for ALK-positive patients, which has shown significant efficacy in ROS1-positive patients as well [187,204]. Clinical trials have reported an ORR of 70–80% and a PFS of approximately 19 months. Despite its efficacy, it has been observed that some patients develop resistance to this drug, leading to tumor recurrence. Resistance to Crizotinib arises due to acquired secondary point mutations [205,206,207], establishing the urgent need to develop new therapies for carriers of this mutation. Entrectinib, a second-generation drug, was subsequently developed in 2016 and immediately showed promising results in clinical studies, recording an overall ORR of 77%, with a median duration of response of 24.6 months [208,209]. Additionally, it demonstrated efficacy in treating brain metastases [210], making it a viable alternative for the treatment of ROS1-positive patients. Along with Entrectinib, Lorlatinib, a third-generation TKI, has shown excellent results in terms of ORR, median PFS, and treatment of brain metastases [211,212,213]. Numerous clinical trials are ongoing to test the efficacy and safety of combined therapies for ROS1-positive tumors, aiming to address emerging resistances, improve survival, and enhance the quality of life for patients. A schematic representation of the available therapies for the well-known lung cancer-involved genes is shown in Table 2.

Despite the availability and use of assigned drugs for common biomarkers of lung cancer, research in this field continues unabated. Owing to the growing understanding of the genetic landscape of lung cancer, new clinical studies are ongoing to assess the efficacy of experimental drugs on less common but equally significant biomarkers (Table 3). This new wave of targeted therapies represents a breakthrough in the fight against lung cancer, offering personalized treatment with fewer side effects compared to traditional ones.

### 3.2. Immunotherapy

The application of immune checkpoint inhibitors (ICIs) has dramatically changed the therapeutic landscape for lung cancer, particularly NSCLC. The United States Food and Drug Administration (FDA) has approved ICIs across three distinct classes: PD-1 inhibitors (nivolumab, pembrolizumab, cemiplimab), PD-L1 inhibitors (atezolizumab, durvalumab, avelumab), and the CTLA-4 inhibitor (ipilimumab). These agents have proven particularly effective when combined with chemotherapy, which addresses tumor and microenvironment heterogeneity and underscores the importance of predictive biomarkers for therapeutic decisions to enhance patient outcomes.

Nivolumab, a fully human IgG4 monoclonal antibody, targets the PD-1 receptor, thereby enhancing effector T-cell populations through induction or expansion, while simultaneously suppressing PD-1 receptor expression on these cells [214]. This mechanism mitigates the inhibitory signal that regulates immune response [215]. As the first humanized monoclonal antibody against PD-1 to receive FDA approval [216], nivolumab marks a significant advancement. It was also the initial agent evaluated in clinical trials for non-small cell lung cancer. Extensive studies have investigated nivolumab across various therapeutic contexts for NSCLC, including neoadjuvant (before surgery), perioperative (around the time of surgery), and consolidation therapies (to maintain remission) [217,218,219]. The result of the NCT01642004 phase III study demonstrated that nivolumab significantly improved median overall survival (9.2 months) compared to docetaxel (6.0 months) and the response rate was higher in the nivolumab group (20%) compared to the docetaxel group (9%) [220]. Moreover, a phase II/III study comparing nivolumab monotherapy to nivolumab plus docetaxel for previously treated advanced NSCLC patients found that the combination therapy resulted in a significantly longer median PFS (3.1 months versus 6.7 months) and median OS (14.7 months vs. 23.1 months), despite an increase in side effects [221]. Additionally, an ongoing phase 2 study explored the dual targeting of PD-1 and LAG-3 with nivolumab and relatlimab before lung cancer surgery, showing feasibility and safety, with high rates of curative resection and promising disease-free and overall survival rates at 12 months [222]. However, not all results have been uniformly positive. The NCT02864251 trial evaluated nivolumab plus chemotherapy versus chemotherapy alone in patients with *EGFR*-mutated NSCLC after progression on EGFR tyrosine kinase inhibitors and found no significant improvement in PFS with the combination therapy, although a trend favoring nivolumab was observed in specific subgroups [223]. Overall, while nivolumab demonstrates substantial benefits over chemotherapy alone in various NSCLC settings, its efficacy can vary based on specific patient subgroups and treatment regimens.

Pembrolizumab is an IgG4 monoclonal antibody that binds to the PD-1 receptor, inhibiting its interaction with PD-L1 and PD-L2. According to the NCT01295827 study, pembrolizumab monotherapy demonstrated durable antitumor activity and high five-year OS rates, particularly in patients with a PD-L1 tumor proportion score of 50% or greater, showing a five-year OS rate exceeding 25% [224]. In the NCT01905657 study, pembrolizumab showed significant OS improvement over docetaxel, particularly in patients with high PD-L1 expression, where median OS was 14.9 months compared to 8.2 months with docetaxel [225]. The safety profile was tolerable, with minimal evidence of late-onset or new toxicity. The NCT02775435 trial, focused on previously untreated metastatic squamous non-small cell lung cancer patients, showing improved OS and PFS with pembrolizumab plus chemotherapy compared to placebo plus chemotherapy, with manageable toxicity and a 5-year OS rate of 18.4% [226]. Moreover, the 5-year results from the phase III NCT02220894 study highlighted the sustained efficacy of pembrolizumab as a first-line treatment for locally advanced or metastatic NSCLC without EGFR or ALK alterations in all PD-L1 tumor proportion score (TPS) groups compared to chemotherapy [227]. The efficacy of pembrolizumab was also confirmed in different real-world studies [228,229]. Ongoing studies are also examining the potential of combining pembrolizumab with other immuno-oncology agents, such as epacadostat, an indoleamine 2,3-dioxygenase inhibitor to unlock immunosuppression in the tumor microenvironment [230]. Despite these promising results from clinical trials, the effectiveness and safety of pembrolizumab in routine practice continue to be areas of active investigation.

Cemiplimab, an anti–programmed cell death-1 (PD-1) monoclonal antibody has been approved for use in combination with chemotherapy as a first-line treatment for patients with advanced NSCLC, regardless of PD-L1 expression levels. This combination therapy has been recommended for adults with NSCLC whose tumors lack *EGFR*, *ALK*, or *ROS1* aberrations [231]. NCT03088540 trials reported that cemiplimab improves overall survival and progression-free survival compared with chemotherapy in patients with advanced non-small cell lung cancer with PD-L1 of at least 50%, providing a potential new treatment option for these patients [232]. Instead, NCT03409614 trials demonstrated that cemiplimab combined with chemotherapy significantly improved overall survival and progression-free survival compared to chemotherapy alone [233]. Additionally, ongoing studies are exploring the efficacy of combining cemiplimab with other immune-oncology agents like fianlimab (anti–LAG-3) to potentially enhance treatment outcomes further [234]. 

Atezolizumab is a humanized IgG1 monoclonal antibody that blocks the interaction of PD-L1 with both PD-1 and B7.1. The NCT02409342 trial evaluated the efficacy of atezolizumab as a first-line treatment for patients with locally advanced or metastatic NSCLC who had not received prior chemotherapy. This trial specifically focused on patients with PD-L1 expression levels of ≥1% on tumor cells or immune cells, excluding those with *EGFR* mutations or *ALK* translocations. The trial demonstrated that atezolizumab is an effective and safer first-line treatment option for NSCLC patients with high PD-L1 expression, providing a significant survival benefit over traditional chemotherapy [235]. However, a randomized phase III trial proved that the combination of atezolizumab and cabozantinib (a tyrosine kinase inhibitor) did not significantly improve overall survival compared to docetaxel in patients with metastatic NSCLC after a checkpoint inhibitor and chemotherapy treatment [236]. In extensive stage SCLC, the combination of atezolizumab with platinum-based chemotherapy has become the standard first-line treatment, showing improved progression-free survival and overall survival compared to chemotherapy alone [237]. Atezolizumab has also been investigated in combination with pirfenidone to target cancer-associated fibroblasts, potentially enhancing efficacy in checkpoint inhibitor-resistant cases. Preliminary results from a phase I/II trial indicated that this combination was well tolerated and showed some efficacy in recurrent NSCLC [238]. Atezolizumab, combined with tiragolumab, is currently under investigation in a randomized phase II trial for unresectable stage III NSCLC both prior to and post definitive chemoradiation [239]. This strategy has the potential to expand the access to immunotherapy for a larger number of patients and enhance outcomes in this clinical context.

Durvalumab, a PD-L1 checkpoint inhibitor, has demonstrated significant promise in the treatment of lung cancer, especially in combination with chemotherapy. The NCT02453282 phase III study, which compared durvalumab monotherapy or durvalumab in combination with tremelimumab (an anti-CTLA-4 antibody) against platinum-based chemotherapy as first-line treatment for advanced NSCLC, did not meet its primary endpoints, as neither durvalumab monotherapy nor the combination of durvalumab and tremelimumab showed a statistically significant improvement in overall survival compared to chemotherapy [240]. However, durvalumab showed significant benefit as a maintenance therapy after chemoradiotherapy in patients with unresectable stage III NSCLC [241]. Also, the NCT03164616 phase III study demonstrated that adding durvalumab to chemotherapy significantly improved progression-free survival in patients with metastatic NSCLC, compared to chemotherapy alone. Moreover, the addition of a limited course of tremelimumab to durvalumab and chemotherapy further improved both progression-free and overall survival, offering a new potential first-line treatment option for patients with metastatic NSCLC, despite the combination treatments being associated with a higher incidence of adverse events [242]. These studies suggest that durvalumab could be a valuable addition to the treatment regimen for NSCLC patients.

Ipilimumab, a monoclonal antibody targeting cytotoxic T lymphocyte-associated antigen-4 (CTLA-4), has been explored in combination with other immune checkpoint inhibitors like nivolumab for the treatment of lung cancer, but its use as monotherapy is less common. In NSCLC, the combination therapy has been studied in trials like NCT02477826 and NCT03215706, where it was observed that patients had significantly better PFS and OS, compared with chemotherapy, regardless of PDL1 status [243,244]. Furthermore, the NCT03158129 trial brought attention to the fact that the combination of neoadjuvant nivolumab and ipilimumab demonstrated a significant pathologic response rate of 38%, in contrast to 22% achieved with nivolumab monotherapy [245]. Additionally, this combination resulted in increased rates of pathologic complete response and enhanced infiltration of immune cells in operable NSCLC, and an examination of neoadjuvant chemotherapy, combined with ipilimumab in early-stage NSCLC, demonstrated notable immune stimulation, especially in CD4+ and CD8+ lymphocytes [246]. Overall, the integration of ipilimumab into lung cancer treatment regimens, particularly in combination with nivolumab, has led to improved survival outcomes and represents a significant advancement in the management of NSCLC.

Unfortunately, despite the described clinical benefits, only about 20–60% of treated patients respond to immunotherapy [247]. In addition, immune-related adverse events (irAEs) remain a significant challenge with ICIs, affecting multiple organ systems with varying severity. Common irAEs include thyroid disease, diabetes, pneumonitis, and various autoimmune conditions [248]. Understanding and predicting these toxicities through biomarkers are critical for optimizing patient management and ensuring the safe administration of ICIs. Biomarkers like PD-L1 expression, tumor mutation burden (TMB), and microsatellite instability-high/mismatch repair-deficient (MSI-H/dMMR) status are shown to be effective in forecasting antitumor effectiveness and potential toxicities of ICIs [249,250,251].

Programmed death-ligand 1 (PD-L1) plays a crucial role in the immune evasion mechanisms of cancer cells and has emerged as a significant therapeutic target and biomarker in NSCLC. The expression level of PD-L1 significantly influences the efficacy of ICIs) such as nivolumab and pembrolizumab [214,252]. High PD-L1 expression (≥50%) has been established as a criterion for administering pembrolizumab monotherapy, now considered the first-line treatment for metastatic NSCLC [252]. Detecting PD-L1 expression in lung cancer involves several methodologies, primarily focusing on immunohistochemistry (IHC) [253] and liquid biopsy techniques [254]. IHC is a widely used method where tissue samples, such as those obtained from surgical resections or biopsies, are stained with specific antibodies against PD-L1. Liquid biopsy, particularly the analysis of circulating tumor cells (CTCs), is another emerging method. Techniques like EpCAM-coated magnetic beads and microfluidic devices have been used to isolate CTCs from blood samples, with subsequent PD-L1 evaluation through immunofluorescent staining [255]. This method has shown promise, with PD-L1 expression detected in a significant proportion of CTCs from SCLC patients. Advanced techniques like radiogenomics, which integrate radiologic imaging with genomic data through artificial intelligence, are also being developed to non-invasively predict PD-L1 expression and other actionable mutations [256]. A multi-label multi-task deep learning system has shown high accuracy in predicting PD-L1 status from CT images, offering a non-invasive alternative to traditional testing methods [257]. Lastly, the prevalence and clinical significance of PD-L1 expression have been studied in various populations, where PD-L1 positivity was associated with specific genetic mutations and histologic types, underscoring the importance of comprehensive molecular profiling in guiding treatment strategies [258]. Collectively, these methods highlight the diverse and evolving landscape of PD-L1 detection in lung cancer, each with its unique advantages and applications in clinical practice. While PD-L1 expression significantly influences treatment outcomes, the variability in response rates and the challenges in accurate assessment necessitate ongoing research and refinement of detection methodologies. Further studies are required to standardize non-invasive PD-L1 testing and validate its predictive power in clinical settings.

TMB, quantified by the number of mutations per megabase of DNA, has gained prominence as a biomarker, with elevated TMB levels indicating a higher likelihood of producing immunogenic neo-antigens capable of eliciting a T-cell response [259]. The FDA’s approval of pembrolizumab for tumors with TMB exceeding 10 mutations per megabase, based on findings from the NCT02628067 trial, has sparked debate due to the intricate nature of TMB as a biomarker [260]. In the context of SCLC, the trial NCT03083691 suggested that high TMB might predict responses to the combination of nivolumab and ipilimumab [261]. For NSCLC, blood-based TMB (b-TMB) has demonstrated a robust correlation with tissue TMB (t-TMB), supporting its use as a reliable biomarker for monitoring treatment response during concurrent chemoradiotherapy [262]. Yet, TMB’s prognostic value in early-stage NSCLC is less clear, as a meta-analysis found no significant differences in OS or disease-free survival (DFS) between high- and low-TMB groups [263]. Real-world evidence from over 8000 patients spanning 24 cancer types treated with ICIs indicated that higher TMB levels were linked to improved OS, reinforcing TMB’s clinical validity as a predictor of ICI efficacy [264]. Detecting TMB expression involves several sophisticated methods to ensure accuracy and reliability, including whole-exome sequencing (WES) and gene-targeted sequencing [265]. TMB testing is typically performed using next-generation sequencing approaches, which range from whole-genome sequencing to WES and large targeted gene panels. WES of tumor versus germline DNA is considered a reference standard, although various commercial gene panel assays and laboratory-developed tests are used [264], differences in gene coverage, in types of mutations included and in computational pipelines to estimate TMB can lead to variability among different laboratories, so their standardization is needed [266]. In the coming years, the assessment of tumor mutational burden may become fundamental in immuno-oncology. However, its application in routine clinical practice requires further optimization.

Deficient DNA mismatch repair (dMMR) refers to the failure of the cellular mechanism responsible for correcting errors that occur during DNA replication. This system involves several key proteins, including MutL protein homolog 1 (MLH1), postmeiotic segregation increased 2 (PMS2), MutS homolog 2 (MSH2), and MutS homolog 6 (MSH6), which work together to recognize and repair mismatches in the DNA sequence. This complex is crucial for maintaining genomic stability by identifying and repairing base-to-base mismatches and insertion–deletion loops that arise during DNA synthesis [267]. dMMR and microsatellite instability high (MSI-H) are emerging as pivotal biomarkers for the efficacy of ICIs across various cancers, but implications in lung cancer remain less well defined [268]. Tumors exhibiting MSI-H are predisposed to robust responses to ICIs, attributed to their elevated mutational burden that promotes neoantigen formation and subsequent immune detection [269]. However, not all dMMR/MSI-H lung cancer patients experience benefit from ICIs [270]. This resistance mechanism may be explained by the fact that MMR-deficient cells can evade apoptosis, a critical pathway for the efficacy of many anticancer therapies, including ICIs [271]. Various advanced techniques have been developed to enhance the sensitivity and specificity to dMMR detection, such as polymerase chain reaction and next-generation sequencing, in colorectal and endometrial cancers [272]. Advancements in deep learning (DL) now enable the prediction of microsatellite instability and deficient mismatch repair status directly from digitized hematoxylin and eosin histopathology slides with high accuracy [273]. These techniques, characterized by high sensitivity, specificity, and simplified protocols, can be adapted for the detection of dMMR to evaluate the efficacy of immune checkpoint inhibitors, ensuring accurate and reliable results in clinical diagnostics.

Peripheral blood for circulating tumor DNA (ctDNA) is emerging as a promising prognostic and predictive biomarker for immune checkpoint inhibitor therapy. Sequential ctDNA studies have demonstrated the ability to detect patients who respond to ICI therapy, including those with NSCLC. A study conducted by Anagnostou et al. demonstrated the correlation between ctDNA response and survival outcomes in NSCLC patients treated with pembrolizumab is significant. Patients with lower ctDNA levels or those whose ctDNA levels decrease during treatment tend to have better survival outcomes [274]. Another study showed that high ctDNA plasma levels are associated with early PD in NSCLC treated with nivolumab [275]. Moreover, a machine learning model was developed that uses longitudinal data of circulating tumor DNA (ctDNA) dynamics to predict survival in NSCLC patients treated in combination with immune checkpoint inhibitors and chemotherapy (chemo-ICI) [276]. In addition to measuring the overall ctDNA burden, further characterization of ctDNA can reveal tumor-specific factors, such as tumor mutational burden and microsatellite instability that influence responses or resistance to immunotherapy [277]. Current methods to measure ctDNA in liquid biopsies involve a variety of advanced techniques aimed at enhancing the precision and utility of cancer diagnostics and monitoring, like RT-PCR, digital PCR (dPCR), mass spectrometry, next-generation sequencing (NGS), and hybrid sequencing (NanoString) [278]. However, it is necessary to adopt specific practical recommendations for ctDNA application to be used routinely in clinical practice to genotype advanced tumors and to be able to choose targeted therapy [279].

The response of non-small cell lung cancer with driver mutations to immune checkpoint inhibitors (ICIs) has been extensively studied due to the varying outcomes seen across different genetic profiles. ICIs have significantly changed the treatment landscape for NSCLC, though their effectiveness in patients with specific molecular alterations remains inconsistent [280]. For instance, patients with EGFR mutations often show poor responses to ICIs. This is likely due to the low immunogenicity of tumors with single driver mutations and the intricate interactions between oncogenic pathways and immune evasion mechanisms [281]. Conversely, NSCLC patients with KRAS mutations generally respond more favorably to ICIs, likely due to higher immunogenicity and PD-L1 expression in these tumors [282]. Research has demonstrated that combining ICIs with chemotherapy (CT-IO) can improve outcomes, especially in patients with novel driver alterations such as MET exon 14 skipping, BRAF mutations, RET rearrangements, and HER2 mutations, compared to ICI monotherapy [283], but with other driver mutations like PIK3CA and STK11, is limited [284]. The integration of molecular diagnostics, such as next-generation sequencing (NGS), is essential for identifying actionable mutations and guiding personalized treatment strategies [285]. Despite these advancements, the optimal sequencing and combination of ICIs, chemotherapy, and targeted therapies are still under investigation to maximize patient outcomes, particularly in never-smoking patients who are more likely to harbor actionable driver mutations [286]. Overall, while ICIs have transformed NSCLC treatment, their efficacy in patients with specific driver alterations varies, highlighting the need for further research to refine therapeutic approaches and improve clinical outcomes.

Several single nucleotide polymorphisms (SNPs) have been identified as influential in determining the efficacy of nivolumab and pembrolizumab across various cancers. In NSCLC patients treated with nivolumab, the CD274 (PD-L1) rs2282055 and rs4143815 SNPs correlated with better ORR and PFS [287]. Specifically, the G allele of rs2282055 and the C/C and C/G genotypes of rs4143815 were linked to significantly better clinical responses compared to alternative alleles [262]. However, another study on NSCLC patients treated with nivolumab found the PDCD1 (PD1) 804C>T SNP was associated with reduced odds of any grade treatment-related toxicities, though this result lacked validation in a subsequent cohort, casting uncertainty on its clinical relevance in predicting toxicity [288]. Another SNP-candidate study performed on a Caucasian cohort of 44 patients tried to validate the predictive role of rs822336, rs2282055, and rs4143815 PD-L1 SNPs in advanced NSCLC patients treated with ICIs. T/T genotype in rs2282055 was slightly associated with longer PFS (*p* = 0.08) and OS (*p* = 0.09), compared to G/T and G/G genotypes. In contrast, patients carrying C/C genotype in rs822336 were significantly associated with better ORR (*p* = 0.004), PFS (*p* = 0.003), and OS (*p* = 0.002), compared to those carrying G/C and G/G genotypes [289]. Genetic factors linked to autoimmune diseases, particularly HLA genes, have been associated with various conditions, highlighting the complex genetic basis of autoimmune disorders. For instance, the HLA class II molecule HLA-DRA has been identified as a marker for immuno-hot tumors, predicting therapeutic responses to anti-PD-1 immunotherapy in NSCLC [290]. Additionally, in patients with advanced solid tumors, including lung cancer, treated with bempegaldesleukin plus nivolumab, certain KIR/KIR-ligand genotypes, such as inhibitory KIR2DL2 and its ligand HLA-C1, were associated with greater tumor shrinkage and improved PFS, suggesting that these genetic factors may also influence responses to nivolumab-based therapies [291].

The integration of biomarkers such as PD-L1 expression, TMB, dMMR/MSI-H status, ctDNA, driver mutations, and SNPs is essential for predicting responses to ICIs in lung cancer. The genetic biomarkers described are just some of those that possibly can be used to predict the efficacy and side effects of ICIs. Research is still ongoing, but many others that are not genetic ones but are particularly promising have already been reported in the literature, such as tumor-infiltrating lymphocytes (TILs) [292], serum pro-inflammatory cytokines [293], and gut microbiome [294]. Advanced detection methods and comprehensive molecular profiling are necessary to refine these biomarkers and improve patient outcomes. Further research is required to standardize non-invasive testing and validate the predictive power of these biomarkers in clinical settings. By enhancing our understanding of these biomarkers and their measurement techniques, personalized cancer therapy can be more effectively tailored to individual patient needs, optimizing treatment efficacy and minimizing adverse effects.

### 3.3. Emerging Therapies 

The introduction of CRISPR-Cas9 gene-editing technology represents a significant advancement in the field of molecular biology and genetic editing. This enables interventions on specific genomic alterations that have the potential to influence lung cancer treatment outcomes, with the potential to substantially improve patient outcomes. The ability to precisely modify the DNA of lung cancer cells using CRISPR-Cas9 offers new opportunities for targeted therapies, overcoming drug resistance, and potentially curing the disease.

Despite the significant progress in CRISPR-Cas9 delivery strategies, numerous challenges remain to be addressed. It is crucial to optimize delivery systems to ensure that the CRISPR-Cas9 complex effectively reaches target cells. Additionally, improving specificity is essential to minimize off-target effects and ensure treatment safety. Equally important is addressing immunogenicity issues, which could limit treatment efficacy, and managing the regulatory and ethical considerations associated with the use of this technology [295].

In this context, ongoing and future clinical studies play a critical role, providing essential data on the feasibility, safety, and therapeutic potential of CRISPR-Cas9-based treatments. These studies will not only facilitate regulatory approval but also provide valuable insights that will guide the optimization of delivery strategies and the selection of target genes.

Delta-like ligand 3 (DLL3) is a member of the delta-like ligand family and plays a crucial role in regulating the Notch pathway, influencing cell differentiation and growth [296]. 3. DLL3 has emerged as a promising therapeutic target in the treatment of SCLC, a notoriously aggressive and difficult-to-treat malignancy. DLL3 is predominantly expressed on SCLC tumor cells and not on normal tissues, making it an ideal target for targeted therapies. In recent years, various therapeutic approaches targeting DLL3 have been developed, including antibody-drug conjugates (ADCs), precision immunotherapies, and radioimmunotherapies [297].

The antibody-drug conjugate Rovalpituzumab Tesirine (Rova-T) was one of the first ADCs to be clinically evaluated. Although initial clinical trials showed promising results, with significant responses in some patients, subsequent studies revealed limited efficacy and high toxicity, leading to the discontinuation of its development. This highlighted the need for further optimization in ADC design and patient selection to enhance therapeutic efficacy [296,297].

In parallel, immunotherapeutic approaches targeting DLL3 to enhance the immune response against SCLC have been explored. Technologies such as CAR-T cells engineered to recognize DLL3 have shown potential in preclinical stages; however, their clinical translation requires further investigation to address safety and efficacy challenges [296,297].

Finally, radioimmunotherapy, which combines the specificity of antibodies with the potency of radiation, is emerging as another innovative strategy. This technique could allow for the selective targeting of DLL3-expressing SCLC tumor cells while minimizing damage to healthy tissue [296].

In summary, while DLL3-based approaches for the treatment of SCLC are still in the developmental phase, current research offers promising prospects. Further studies are needed to improve the safety and efficacy of these therapies, with the goal of establishing DLL3 as a key target in the fight against SCLC.

The next generation of treatment for several neoplasms also includes chimeric antigen receptor (CAR) T cells therapy. CAR-T cells are engineered T lymphocytes derived from allogeneic healthy donors or from the same patient, modified to express a receptor directed against the tumor cell surface antigens, in order to kill them [298].

The major challenge in CAR-T treatment is the target antigen choice, that needs to be highly specific, expressed and stable [299] such as, for lung cancers, the epidermal growth factor receptor (EGFR) [300], mucin 1, cell surface associated (MUC1) [301], the receptor tyrosine kinase like orphan receptor 1 (ROR1) [302], mesothelin (MSLN) [303]. Among these, EGFR and MSLN seem to be the more promising targets compared to others: indeed, they showed higher antigen specificity and lower on-target, off-tumor toxicity concern, i.e., the potential healthy organ toxicity, due to the expression of these targets in tissues other than tumor [304]. 

Up to this date, 29 different phase I/II clinical trial led by Chinese (i.e., NCT03525782, NCT05060796, NCT05117138,NCT02876978) and 15 (i.e., NCT02706392, NCT05274451, NCT05239143) led by American hospitals are still ongoing; a study closed in 2019 (NCT02706392) demonstrated that the already existing efficacy of CAR-T therapy in patients with lung cancer can be improved with the combination with Ox/Cy and anti-PD-L1 [305] showing that that the use of CAR-T therapy led to really promising results [306]. Another clinical trial (NCT04025216) evaluated the effectiveness against Tn-MUC1; it was closed in 2023 but the results were not yet presented. To the best of our knowledge, only one registered Australian/German phase I/II clinical trial (NCT04503278) evaluating the effectiveness of CLDN6 CAR-T therapy on solid tumors, with a focus also on NSCLC, is still on recruitment. 

Although CAR-T therapy was first approved in 2017 and 2018 by the American Food and Drug Administration and the European Medicines Agency, respectively, the use of CAR-T cell therapy for solid tumors, including lung cancer, is still debated [307,308] and research is still ongoing. In particular, the clinical applicability of CAR-T therapy in lung cancer, as well as other solid tumors, is hindered by challenges such as inadequate T cell infiltration into the solid tumor microenvironment, T-cell exhaustion, and the variability in tumor antigen expression. 

To overcome some limitations of CAR-T therapy for the treatment of solid tumors, innovative approaches, such as the CAR-M or CAR-NK cells therapy, are under investigation, but these strategies are still in early stages of development. So far, only three registered trials (NCT06454890, NCT05507593, NCT02839954) are focused on lung cancer, and they are not yet recruiting. Nevertheless, CAR-M and CAR-NK are a very promising field, as their particular characteristics allow them to be better utilized in solid tumors [301].

## 4. Challenges and Future Prospects

Lung cancer represents one of the leading causes of mortality worldwide, with a generally poor prognosis due to late diagnosis and the aggressive nature of the disease. In recent decades, oncological research has made significant progress, paving the way for new therapeutic strategies based on the personalization of treatments. This approach aims to optimize the effectiveness of therapies and reduce side effects by considering the specific characteristics of the tumor and the patient.

The understanding of the molecular bases of lung cancer has revolutionized clinical oncology. Genetic studies have identified numerous mutations and genetic alterations that drive the growth and proliferation of tumor cells. The identification of such mutations through molecular tests has become crucial for treatment selection. In this context, targeted therapies and immunotherapy have represented a significant breakthrough in the management of lung cancer. However, many patients have developed acquired resistance to treatments. Acquired resistance occurs when tumor cells, initially sensitive to treatment, evolve mechanisms that render them refractory. This phenomenon can manifest through various mechanisms, including secondary mutations, activation of alternative signaling pathways, and phenotypic modifications of the tumor. To date, this represents a crucial obstacle but also the starting point for personalized medicine. Precision medicine indeed goes beyond the mere identification of genetic mutations, also including the analysis of the tumor microenvironment, gene expression profiles, and other biomarkers. Advanced techniques such as liquid biopsy, which allows for the analysis of circulating tumor DNA in the blood, are emerging as promising tools for monitoring disease progression and treatment efficacy in real time. The personalization of therapeutic choices requires a multidisciplinary approach involving oncologists, pathologists, radiologists, and geneticists. Every patient is unique, and therapeutic decisions must consider various factors, including age, general health status, comorbidities, and personal preferences. Continuous dialogue between the medical team and the patient is essential to ensure that therapeutic choices are aligned with care objectives and quality of life. In addition, another obstacle to overcome is the limited access to innovative therapies due to economic and logistical factors. For this reason, it is essential to fund ongoing research to identify new therapeutic targets, improve understanding of the interactions between cancer and the immune system, and ensure access to treatment for all types of patients.

In the future, the integration of omics data (genomics, proteomics, metabolomics) and the use of artificial intelligence to analyze large volumes of clinical data could further revolutionize the personalization of care. The development of more accurate predictive models will allow for the anticipation of treatment responses and the dynamic adaptation of therapeutic strategies.

Another crucial aspect in the management of lung cancer is prevention and early diagnosis. Screening programs, such as low-dose computed tomography (LDCT), have proven effective in reducing mortality among high-risk individuals, such as smokers. Moreover, awareness campaigns about the risks associated with smoking and exposure to harmful substances, such as asbestos, are essential for the primary prevention of the disease. Immunotherapy, in particular, has opened new avenues in the treatment of lung cancer. Treatments with immune checkpoint inhibitors, such as anti-PD-1 and anti-PD-L1 antibodies, have shown promising results, increasing survival rates in patients with advanced disease. However, the response to immunotherapy varies significantly among patients, and ongoing research is focused on identifying predictive biomarkers to better select candidates for this type of treatment. Finally, international collaboration and the sharing of clinical data through global research networks can accelerate discoveries and improve care standards worldwide. Ensuring equitable access to new technologies and innovative treatments remains a challenge, requiring global health policies aimed at reducing disparities in healthcare.

## 5. Conclusions

This review highlights the intricate and complex nature of treatment based on genetic data in lung cancer patients, underscoring the importance of evaluating and monitoring the cancer genetic landscape. Genetic discoveries have transformed the approach to lung cancer treatment, leading to targeted, personalized therapies that improve patient survival and quality of life. However, challenges such as therapeutic resistance and the need for more precise customization require further research and clinical innovation. The future of lung cancer therapy lies in the integration of advanced genomics with clinical practice to develop increasingly effective treatments.

## Figures and Tables

**Table 1:** Summary of lung cancer genetic mutations with the frequency of their detection, the metabolic pathway in which they are involved, and for which lung cancer subtype they are typical.

Gene	Frequency [%]	Metabolic Pathways	Type of Lung Cancer
EGFR	30–40	PI3K/AKT, RAS/RAF/MEK/ERK	Adenocarcinoma Squamous cell carcinomas Large cell carcinoma
KRAS	20–30	MAPK, PI3K-AKT	Adenocarcinoma Large cell carcinoma
ALK	3–7	STAT3, mTOR, PI3K, Ras and MEK.	Adenocarcinoma Squamous cell carcinomas Large cell carcinoma
BRAF	1–3	MAPK/ERK	Adenocarcinoma Large cell carcinoma
ROS1	1–2	JAK/STAT, PI3K/AKT, and MAPK/ERK	Adenocarcinoma Squamous cell carcinomas Large cell carcinoma
PD-L1	20–30	JAK/STAT, PI3K/Akt/mTOR, MAPK/ERK, NF-κB e TGF-β	Adenocarcinoma Squamous cell carcinomas
MET	3–4 1–6 0.2–0.3	PI3K/AKT and MAPK/ERK	Adenocarcinoma Large cell carcinoma
RET	1–2	RAS/MAPK/ERK, PI3K/AKT	Adenocarcinoma Large cell carcinoma
NTRK	0.1–0.2	PIK3/PLCγ/MAPK	Adenocarcinoma Large cell carcinoma
PIK3CA	2–4	PI3K/AKT/mTOR	Adenocarcinoma Squamous cell carcinomas Large cell carcinoma
HER2	1–3	MAPK, PI3K/AKT, protein kinase C and STAT,	Adenocarcinoma Squamous cell carcinomas Large cell carcinoma
STK11	20–30	Dysfunction of the AMPK pathway	Adenocarcinoma Squamous cell carcinomas Large cell carcinoma

**Table 2:** Well-known genes involved in lung cancer and current therapies.

Gene	Molecular Alteration	Locus	Mutational Hotspot	Drug
EGFR	Mutation	7p11.2	Deletion in exon 19 Substitution in exon 21 (L858R)	Gefitinib Erlotinib Afatinib Osimertinib
KRAS	Mutation	12p12.1	Substitution in exon 12 (G12C), Substitution in exon 13 (G12D) Substitution in exon 61 (G12V)	Sotorasib Adagrasib
ALK	Variable chromosome rearrangement	2p23.2-p23.1	Fusion EML4-ALK, KIF5B-ALK, TFG-ALK, KLC1-ALK	Crizotinib Ceritinib Alectinib Brigatinib Lorlatinib
BRAF	Mutation	7q34	Sostitution V600E, V600D, V600K, V600R K601N/E, L597V, G464V, G469V/R/A, G466V/A, N581S, D594N/G, G596R	Vemurafenib Dabrafenib Trametinib
ROS1	Variable chromosome rearrangement	6q22.1	Fusion CD74-ROS1, SLC34A2-ROS1, TPM3-ROS1, SDC4-ROS1	Vemurafenib Dabrafenib Trametinib

**Table 3:** Emerging genes involved in lung cancer and current therapies.

Gene	Molecular Alteration	Locus	Mutational Hotspot	Drug
PD-L1	Mutation Amplification	9p24.1	Substitution G1268C	Nivolumab Pembrolizumab Cemiplimab Durvalumab Atezolizumab Avelumab
MET	Amplification Mutation Chromosome rearrangement	7q31.2	METex14 Fusion KIF5B-MET	Crizotinib Tepotinib Capmatinib Bozitnib Glumetinib
RET	Variabel chromosome rearrangement Mutation	10q11.21	Fusion KIF5B-RET, CCDC6-RET, NCOA4-RET, TRIM33-RET, CUX1-RET	Pralsetinib Selpercatinib
NTRK	Gene fusions	13q31.1 13q31.2 Xq27.3 3q26.1	Fusion SQSTM1-NTRK1, RFWD2-NTRK1, CD74-NTRK1, TRIM 24–NTRK2	Entrectinib Larotrectinib Repotrectinib
PIK3CA	Mutation Amplification	3q26.32	Substitution in exon 9 (E545K/E542K) Substitution in exon 20 (H1047R/H1047L)	Gedatolisib Idelalisib
HER2	Mutation Amplification Overexpression	17q12	Insertion in exon 20 (A775_G776insYVMA)	Ado-trastuzumab emtansine Mobocertinib Poziotinib
STK11	Mutation Inactivation	19p13.3	Deletion of 19p13.3	Atezolizumab Pembrolizumab Nivolumab

## Data Availability

Not applicable.
